# Correlation between body composition and disease severity in patients with chronic obstructive pulmonary disease

**DOI:** 10.3389/fmed.2024.1304384

**Published:** 2024-03-14

**Authors:** Xiaohan Jin, Yu Yang, Guangmei Chen, Yue Shao, Chang Liu, Rong Li, Yazhuo Liu, Lichuan Zhang

**Affiliations:** ^1^Department of Respiratory Medicine, Affiliated Zhongshan Hospital of Dalian University, Dalian, China; ^2^Department of Respiratory Medicine, Shandong Second Rehabilitation Hospital, Tai'an, China; ^3^Department of Respiratory Medicine, Panjiang General Hospital, Panzhou, China

**Keywords:** chronic obstructive pulmonary disease, bioelectrical impedance analysis, body composition, comprehensive assessment, nutrition

## Abstract

**Background:**

Body composition changes are important extrapulmonary manifestations in chronic obstructive pulmonary disease (COPD) patients. This study aimed to investigate the characteristics of body composition in patients with COPD, and its correlation with disease severity.

**Methods:**

A total of 105 COPD patients admitted to Zhongshan Hospital affiliated to Dalian University, from May 1, 2021 to January 31, 2023, were included as the COPD group, and 105 subjects without COPD were enrolled as the control group during the same period. According to the Global Initiative for Chronic Obstructive Lung Disease (GOLD) comprehensive assessment indicators, COPD patients were divided into groups: the degree of pulmonary function airflow limitation was grouped according to FEV1%pred; clinical symptoms were grouped according to mMRC scores and CAT scores; the risk of acute exacerbation was divided into low risk and high risk groups. Body composition was measured by bioelectrical impedance analysis (BIA).

**Results:**

(1) Concerning body composition, the body mass index (BMI), fat-free mass index (FFMI), and angle of phase (PhA) of COPD patients were lower than those of the control group. Extracellular water-to-total body water ratio (ECW/TBW) and extra-to-intracellular water ratio (ECW/ICW) were higher than those of the control group, and the difference was statistically significant (*p* < 0.05). (2) There were differences in body composition among COPD patients with different severity of disease: FFMI and PhA in the mild/moderate airflow limitation group were higher than those in the severe/very severe airflow limitation group. According to mMRC scores classification, the FFMI and PhA of the less symptomatic group were higher than those of the more symptomatic group, and ECW/TBW and ECW/ICW were lower than those of the more symptomatic group. According to CAT scores classification, FFMI and PhA in the mild/moderate disease group were higher than those in the severe/very severe disease group. The FFMI of the low-risk group was higher than that of the high-risk group, and ECW/TBW was lower than that of the high risk group. (3) Correlation analysis between body composition and disease severity indicators showed that FFMI and PhA were negatively correlated with mMRC scores and CAT scores, and positively correlated with FEV1%pred. ECW/TBW ratio and ECW/ICW ratio were positively correlated with mMRC scores and CAT scores, and negatively correlated with FEV1%pred, and the difference was statistically significant (*p* < 0.05).

**Conclusion:**

There are significant differences in body composition between COPD patients and the control group, and there are significant differences in body composition between COPD patients with different severity of disease, with correlations between body composition and severity of disease.

## 1 Introduction

Chronic obstructive pulmonary disease (COPD) is a heterogeneous lung disease characterized by chronic respiratory symptoms (dyspnea, cough, and expectoration). It is the third leading cause of death in the world, and ranks among the top five disease burdens in the world (1). Both the 2021 Chinese guidelines for the diagnosis and treatment of chronic obstructive pulmonary disease (2), and 2023 Global Initiative for Chronic Obstructive Lung Disease (GOLD) (1), emphasize that the severity of COPD in patients needs to be comprehensively evaluated by considering the degree of airflow limitation, clinical symptoms, and risk of acute exacerbations.

The COPD patients are prone to malnutrition due to long-term chronic hypoxia, systemic inflammatory response, and metabolic disorders (3). Body composition changes in patients with COPD include sarcopenia, obesity, and sarcopenic obesity. Malnutrition, an independent predictor of poor prognosis in COPD, leads to impaired lung function, reduced exercise capacity, accelerated disease progression, and increased mortality (4). Thus, in 2014, the European Respiratory Society proposed the concept of COPD based on the measurement of body composition, which was used as the basis of nutritional assessment (5), suggesting that body composition should be an important part of the routine assessment of the nutritional status of COPD patients (5).

Body composition is essential for the assessment of nutritional status of COPD patients. Hydrodensitometry is one of the methods considered to be the gold standard in the analysis of body composition. As this method requires the subjects to be submerged in water, it is not comfortable for COPD patients. Anthropometry is the least expensive method, but presents some disadvantages: it requires specialized personnel and is based upon the principle of a constant body-fat fraction (6). Dual energy X-ray absorptiometry (DEXA) has been suggested as a suitable clinical reference method for the measurement of body composition. However, DEXA is an expensive method and contains a small amount of radiation (7). Based on the conductive characteristics of the human body, bioelectrical impedance analysis (BIA) can provide body composition data by passing a small amount of current through the human body according to the principle of the difference in tissue impedance. It is cheap, convenient, accurate and reliable, and has become the most widely used method to evaluate body composition in clinical practice (8). BIA and anthropometry presented good reliability and correlation with DEXA; the three methods presented satisfactory clinical accuracy (6). There were no significant differences between body composition variables obtained by DEXA and by BIA (9). And, the Asian Working Group for Sarcopenia (AWGS 2019) recommends the use of DEXA or BIA to measure muscle mass (10). In addition, there are methods considered to be reference standards, such as magnetic resonance, dilution techniques and others which are not feasible in clinical practice (11).

Body composition indicators commonly used in nutritional assessment of patients with respiratory diseases include body mass index (BMI), free mass index (FMI), fat-free mass index (FFMI), Intracellular water (ICW), Extracellular water (ECW), and angle of phase (PhA). In the past, BMI was commonly used to evaluate the nutritional status of patients, however, BMI does not clearly reflect changes in muscle mass (12). FFMI is a strong predictor of muscle loss. Therefore, compared with BMI, FFMI is a more sensitive means to assess the nutritional status of a patient (13). In addition to muscle status, hydration status is also important in body composition. As muscle loss is often accompanied by water imbalance (14). The intracellular component of FFM is mostly water, and BIA technology can indirectly and easily measure the water distribution based on the electrical impedance of current conduction in the body (15). The hydration state of cells (contraction or swelling) changes dynamically, and measurement of the hydration state of cells can provide quantitative information about the water content of tissues and organs, as well as a qualitative assessment of cell function (16). The extra-to-intracellular water ratio (ECW/ICW) volume changes with age (17). Especially in the elderly with chronic wasting diseases, the decrease of ICW is more obvious (14), accompanied by a relative or absolute increase in extracellular water (ECW) (18). The ratios ECW/ICW and ECW/TBW can be used as indicators of the hydration status of cells (19). The human body is a complex organism. ECW and ICW can be regarded as resistance, and the cell membrane can be regarded as capacitance because of its voltage difference; the bioelectrical impedance value of the resistance and capacitance together can be considered as a vector. The angle between the direction of the vector and the coordinate axis is the angle of PhA, which reflects the magnitude of the resistance of human cell membranes to alternating current. PhA provides information on the hydration status of the cells, the quality and quantity of the cells, the integrity of the cells, and the prognosis of the cells, and it reflects their functional status, and can be used to screen for nutritional status, and can be used to assess the nutritional status of cells (20). PhA is associated with inflammation, malnutrition and lack of levels of physical activity, and is reduced in disease states such as malignancy, heart failure, cirrhosis, and AIDS (21).

Few studies have reported on the correlation between the above-mentioned body composition characteristics and the assessment of comprehensive indicators of disease status in COPD. The aim of this study was to investigate the body composition characteristics of patients with COPD and their correlation with the severity of the disease, in order to understand the nutritional status of patients with COPD in different disease states in a more comprehensive and in-depth manner, and to lay the foundation for the development of a reasonable and effective individualized comprehensive treatment plan.

## 2 Materials and methods

### 2.1 Study population

This was a single center observational study, performed at the Affiliated Zhongshan Hospital of Dalian University, in Dalian, China, from May 1, 2021 to January 31, 2023.The study included 105 randomly selected COPD patients. During hospitalization, COPD patients were given standardized diagnosis and treatment, including anti-infection, antitussive and expectorant treatment, antiasthmatic treatment, and nutritional support, without rehabilitation treatment. Inclusion criteria included (1) aged ≥18 years, (2) COPD patients diagnosed according to GOLD (2021) (2), and (3) willing to participate in this survey. Exclusion criteria included: (1) known history of significant inflammatory disease other than COPD, such as bronchiectasis or lung abscess, (2) patients with acute or chronic kidney disease, heart failure, cor pulmonale, liver disease, malignant disease, ascites, and visible signs of peripheral oedema, (3) history of mental illness, or alcohol or substance abuse, and (4) a contraindication to BIA including an implanted pacemaker, defibrillator or joint prosthesis. A case–control approach was used. In addition, during the same period, 105 subjects without COPD were selected as the control group.

The study protocol was approved by the Institutional Ethics Committee of the Affiliated Zhongshan Hospital of Dalian University and conducted according to the Declaration of Helsinki. All methods were performed in accordance with the relevant guidelines and regulations. Written informed consent was obtained for analysis of demographics and medical histories.

### 2.2 Data collection

Basic demographic data (age, sex, and occupational history) were obtained together with a structured medical history. Medical history in patients with COPD included history of acute COPD exacerbations, hospitalisations, and routine and/or acute medication use for COPD in the past year.

Data on participants’ clinical symptoms were collected during face-to-face interviews with well-trained personnel, including the score of modified Medical British Research Council (mMRC) and score of COPD assessment test (CAT).

Body weight and height were measured while the participants were barefoot and dressed in light clothing. Weight was measured using a digital weighing scale (BF-220, TANITA, Tokyo, Japan), and height using a wall-mounted stadiometer, and values were rounded to the nearest 0.1 kg and 0.1 cm, respectively.

#### 2.2.1 Pulmonary function tests

In this study, the pulmonary function of all subjects was measured by the same staff using a German Jaeger pulmonary function instrument in the pulmonary function room at the hospital. Following bronchodilator (salbutamol, 200 μg) inhalation, the forced expiratory volume in the first second/forced vital capacity (FEV1/FVC) and forced expiratory volume in 1 s percent predicted value (FEV1%pred) were recorded.

The participants were stratified according to the criteria of the Global Initiative for Chronic Obstructive Lung Disease (2021) (2) for FEV1%pred and FEV1/FVC ratio as follows:

- Mild COPD: FEV1% predicted ≥80%, and FEV1/FVC < 0.7
- Moderate COPD: FEV1% predicted 50–79%, and FEV1/FVC < 0.7
- Severe COPD: FEV1% predicted 30–49%, and FEV1/FVC < 0.7
- Very severe COPD: FEV1% predicted <30%, and FEV1/FVC < 0.7.

#### 2.2.2 Body composition

Body composition was assessed by single-frequency BIA (INBODY-770, Inbody Corporation, South Korea). All body composition measurements were performed in the morning on an empty stomach and no strenuous exercise prior to measurement. The subject stood barefoot on the instrument, contacted the electrode plate with feet, clasped the electrode handle with both hands, pressed both thumb against the electrode point, and spread the arms naturally laterally. The instrument records the relevant indicators of body mass index (BMI), fat mass (FM), fat mass index (FMI), fat-free mass index (FFM), fat-free mass index (FFMI), phase angle (PhA), intracellular water (ICW), extracellular water (ECW), total body water (TBW), extracellular water-to-total body water ratio (ECW/TBW), and extra-to-intracellular water ratio (ECW/ICW).

### 2.3 Statistical analyses

Data are presented as mean ± standard deviation (SD) for normally distributed variables, and as median with interquartile range (IQR) for skew distributed continuous variables. Categorical variables are presented as frequencies.

Data were analyzed using Pearson’s chi-squared test for categorical variables, Student’s *t*-test for normally distributed continuous data, and the Mann–Whitney *U* test for skew distributed parameters was applied for univariate analysis. Pearson’s correlation coefficient or Spearman’s correlation coefficient was used to evaluate the associations between variables. Multiple linear regression model was used to explore the relationship between body composition variables and disease severity (FEV1%pred).

GraphPad Prism 9 was used to analyze and plot data. Statistical analyses were performed using SPSS version 25.0; *p* < 0.05 was considered significant in all analyses.

## 3 Results

### 3.1 Basic clinical characteristics of subjects

In this study, 105 patients with COPD (84 males and 21 females) and 105 controls (75 males and 30 females) were enrolled. Baseline characteristics of the study groups are presented in Table 1.

**Table 1:** Basic clinical characteristics of subjects.

Variable	COPD group (*n* = 105)	Control group (*n* = 105)	*p*-value
Age, years	69.71 ± 9.23	68.06 ± 3.58	0.089
sex			0.148
Male, *n*(%)	84 (80%)	75 (71.40%)	
Female, *n*(%)	21 (20%)	30 (28.60%)	
Current or ex-smoker, *n*(%)	63 (60%)		
Course of disease, (year)	15.1 ± 14. 2		
FEV1/FVC, %	58.42 ± 12.68		
FEV1% pred, %	51.59 ± 18.49		
Previous COPD AEs (exacerbations/year)	1.03 ± 0.77		
In hospital death	0		
The score of mMRC	2.20 ± 1.09		
The score of CAT	21.61 ± 7.56		

Data are reported as the mean (SD) or *n* (%); COPD, chronic obstructive pulmonary disease; FEV1, forced expiratory volume in the first second; FVC, forced vital capacity; AE, acute exacerbation; mMRC, modified medical British research council; CAT, COPD Assessment Test.

### 3.2 Body composition measurements in the COPD group and the control group

**Figure 1:**
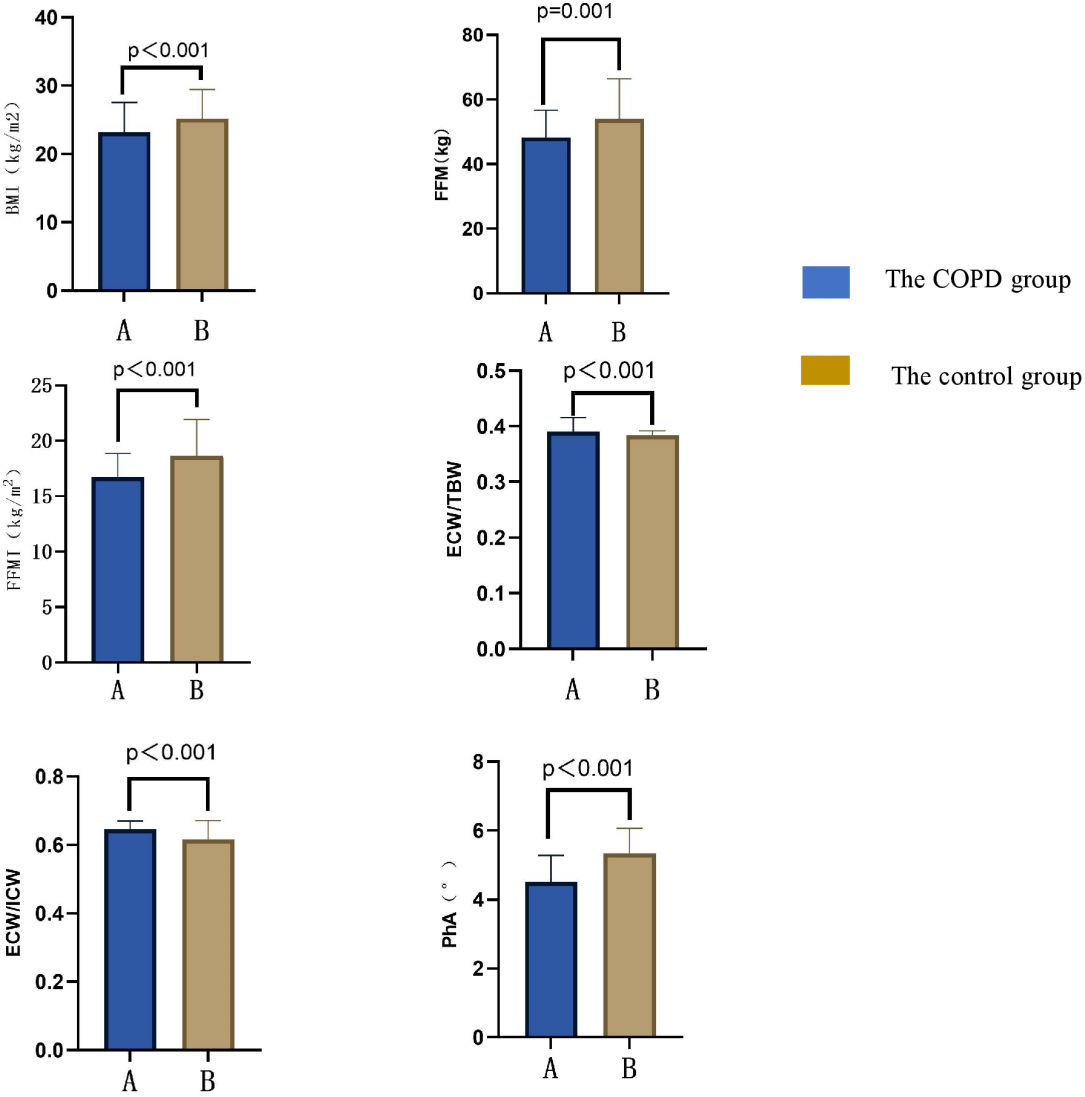
Comparison of body compositions between COPD patients and the control group. BMI, body mass index; FFM, fat free mass; FFMI, fat free mass index; PhA, angle of phase; ICW, intracellular water; ECW, the extracellular water ratio; TBW, total body water.

The results of body composition measurements in the COPD group and the control group are shown in Figure 1. BMI, FFM, FFMI, and PhA in the COPD group were lower than those in the control group (*p* < 0.05); ECW/TBW and ECW/ICW in the COPD group were higher than in the control group (*p* < 0.05). Additional details can be found in Table 2.

**Table 2:** Comparison of body composition between COPD group and the control group.

Variable	The COPD group (*n* = 105)	The control group (*n* = 105)	*P*-value
Height, m	1.70 (0.10)	1.69 ± 0.09	0.531
Weight, kg	66.80 (15.80)	73.06 ± 14.29	0.004
BMI, kg/m^2^	23.22 ± 4.34	25.00 (5.15)	<0.001
FFM, kg	48.70 (9.05)	54.40 (19.35)	0.001
FFMI, kg/m^2^	16.72 ± 2.13	18.64 ± 3.26	<0.001
PhA, °	4.52 ± 0.76	5.34 ± 0.73	<0.001
ICW, L	21.68 ± 3.85	22.70 (8.30)	0.122
ECW, L	14.10 (2.55)	14.10 (4.75)	0.848
TBW, L	36.00 (6.60)	36.60 (13.00)	0.305
ECW/TBW	0.39 (0.01)	0.38 (0.01)	<0.001
ECW/ICW	0.64 (0.03)	0.62 (0.02)	<0.001

BMI, body mass index; FFM, fat free mass; FFMI, fat free mass index; PhA, angle of phase; ICW, intracellular water; ECW, the extracellular water ratio; TBW, total body water.

### 3.3 Stratification analysis

#### 3.3.1 Stratified by the degree of pulmonary function airflow limitation

COPD patients were stratified according to the degree of pulmonary function airflow limitation. A comparison of body composition between the mild/moderate airflow limitation group (FEV1%pred ≥50%) and the severe/very severe airflow limitation group (FEV1%pred < 50%) is shown in Table 3. FFMI and PhA in the mild/moderate airflow limitation group were significantly higher than in the severe/very severe airflow limitation group (*p* < 0.05).

**Table 3:** Comparison of body composition between mild/moderate group and severe/very severe group in COPD patients with airflow limitation.

Variable	The mild/moderate airflow limitation group (*n* = 56)	The severe/very severe airflow limitation group (*n* = 49)	*P*-value
Height, m	1.70 (0.10)	1.71 (0.08)	0.645
Weight, kg	67.95 (14.10)	65.15 ± 14.75	0.141
BMI, kg/m^2^	23.94 ± 4.25	22.40 ± 4.34	0.069
FFM, kg	49.77 ± 8.55	46.72 ± 8.00	0.063
FFMI, kg/m^2^	17.30 ± 2.01	16.18 ± 2.03	0.006
PhA, °	4.70 ± 0.76	4.31 ± 0.71	0.009
ICW, L	22.36 ± 3.95	20.91 ± 3.62	0.052
ECW, L	14.35 (2.65)	13.57 ± 2.37	0.095
TBW, L	36.71 ± 6.33	34.51 ± 5.94	0.070
ECW/TBW	0.39 ± 0.01	0.39 (0.01)	0.191
ECW/ICW	0.64 ± 0.02	0.65 ± 0.03	0.158

BMI, body mass index; FFM, fat free mass; FFMI, fat free mass index; PhA, angle of phase; ICW, intracellular water; ECW, the extracellular water ratio; TBW, total body water.

#### 3.3.2 Stratified by clinical symptoms

##### 3.3.2.1 Stratified by mMRC score

Differences in body composition in patients with different clinical symptoms. Table 4 shows the differences in body composition grouped by mMRC score. FFMI and PhA in the less symptomatic group (mMRC scores: 0–1) were higher than in the more symptomatic group (mMRC scores: 2–4), and ECW/TBW and ECW/ICW were lower than in the more symptomatic group.

**Table 4:** Comparison of body composition between less symptomatic group and more symptomatic group in COPD patients.

Variable	The less symptomatic group (*n* = 31)	The more symptomatic group (*n* = 74)	*P*-value
Height, m	1.70 ± 0.07	1.70 (0.09)	0.424
Weight, kg	72.00 (15.60)	62.80 (14.90)	<0.001
BMI,kg/m^2^	25.42 ± 4.45	22.05 (4.70)	<0.001
FFM,kg	54.12 ± 7.99	45.93 ± 7.36	<0.001
FFMI,kg/m^2^	18.58 ± 1.67	16.02 ± 1.76	<0.001
PhA, °	5.02 ± 0.66	4.31 ± 0.69	<0.001
ICW, L	24.42 ± 3.65	20.54 ± 3.33	<0.001
ECW, L	15.54 ± 2,26	13.33 ± 2.17	<0.001
TBW, L	40.00 ± 5.90	33.89 ± 5.46	<0.001
ECW/TBW	0.38 (0.01)	0.39 (0.01)	0.021
ECW/ICW	0.63 (0.02)	0.65 (0.03)	0.009

BMI, body mass index; FFM, fat free mass; FFMI, fat free mass index; PhA, angle of phase; ICW, intracellular water; ECW, the extracellular water ratio; TBW, total body water.

##### 3.3.2.2 Stratified by CAT score

Table 5 shows the differences in body composition stratified according to CAT scores. BMI, FFM, FFMI, and PhA in the mild/moderate disease group (CAT <20) were higher than those in the severe/very severe disease group (CAT >20) (*p* < 0.05).

**Table 5:** Comparison of body composition between mild/moderate and severe/very severe COPD patients.

Variable	The mild/moderate disease group *n* = 43	The severe/very severe disease group *n* = 62	*P*-value
Height, m	1.70 (0.13)	1.70 (0.08)	0.188
Weight, kg	70.30 (20.00)	63.52 ± 12.07	0.002
BMI(kg/m^2^)	24.82 ± 4.88	22.11 ± 3.57	0.001
FFM,kg	51.96 ± 9.17	45.84 ± 6.84	<0.001
FFMI,kg/m^2^	17.77 ± 2.23	16.09 ± 1.67	<0.001
PhA, °	4.81 ± 0.76	4.31 ± 0.69	0.001
ICW, L	23.39 ± 4.19	20.50 ± 3.12	0.001
ECW, L	14.99 ± 2.60	13.29 ± 2.01	<0.001
TBW, L	38.37 ± 6.77	33.82 ± 5.08	<0.001
ECW/TBW	0.38 (0.01)	0.39 (0.01)	0.120
ECW/ICW	0.64 (0.03)	0.65 (0.03)	0.058

BMI, body mass index; FFM, fat free mass; FFMI, fat free mass index; PhA, angle of phase; ICW, intracellular water; ECW, the extracellular water ratio; TBW, total body water.

#### 3.3.3 Stratified by acute exacerbations

Table 6 shows the characteristics of body composition in the low-risk and high-risk groups for acute exacerbations in patients with COPD. FFMI in the low-risk group (0 or 1 moderate exacerbations and not leading to hospitalization) was higher than in the high-risk group (≥2 moderate exacerbations or ≥1 leading to hospitalization), and ECW/TBW was lower than in the high-risk group.

**Table 6:** Comparison of body composition between low risk group and high risk group of acute exacerbation in COPD patients.

Variable	The low-risk group (*n* = 57)	The high-risk group (*n* = 48)	*P*-value
Height, m	1.70 (0.11)	1.70 (0.07)	0.433
Weight, kg	67.76 ± 14.53	65.30 (16.30)	0.524
BMI(kg/m^2^)	23.43 ± 4.70	22.40 (4.58)	0.378
FFM, kg	49.15 ± 9.27	47.39 ± 7.21	0.287
FFMI, kg/m^2^	17.14 ± 2.34	16.34 ± 1.66	0.048
PhA, °	4.63 ± 0.74	4.38 ± 0.76	0.096
ICW, L	22.09 ± 4.26	21.20 ± 3.28	0.239
ECW, L	14.60 (2.40)	13.78 ± 2.11	0.336
TBW, L	37.10 (6.40)	34.75 (7.20)	0.271
ECW/TBW	0.38 (0.01)	0.39 (0.01)	0.045
ECW/ICW	0.64 (0.03)	0.65 (0.03)	0.073

BMI, body mass index; FFM, fat free mass; FFMI, fat free mass index; PhA, angle of phase; ICW, intracellular water; ECW, the extracellular water ratio; TBW, total body water.

### 3.4 Correlations between disease severity and body composition

**Figure 2:**
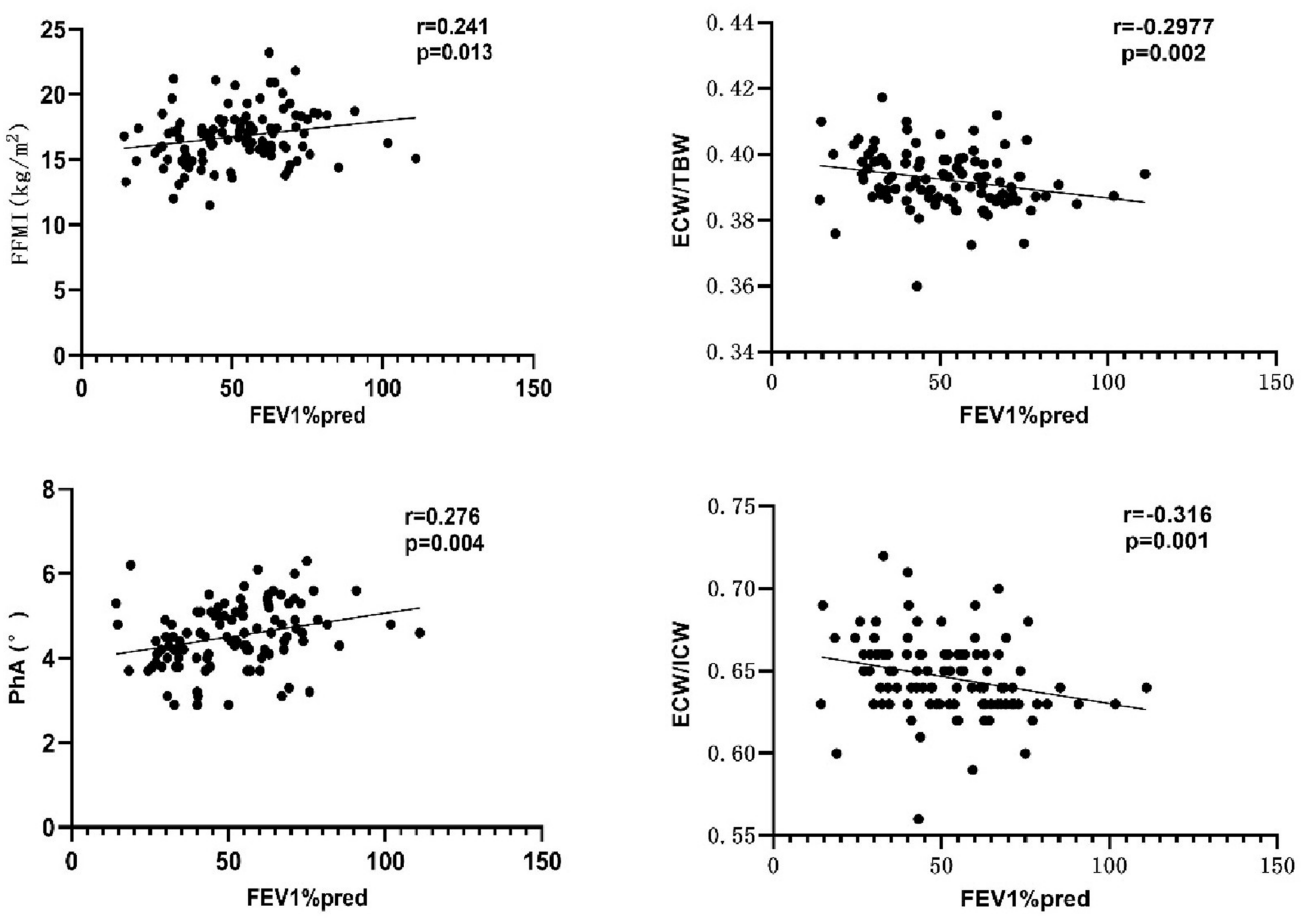
Correlation between FEV1%pred and body composition. PhA, phase angle; FFMI, fat-free body mass index; ECW/TBW, extracellular water-to-total body water ratio; ECW/ICW, extra-to-intracellular water ratio.

As shown in Figure 2, FEV1%pred was positively correlated with FFMI and PhA, and negatively correlated with ECW/TBW and ECW/ICW. Additional details can be found in Table 7. Both mMRC scores and CAT scores were negatively correlated with FFMI and PhA, and positively correlated with ECW/TBW and ECW/ICW (*p* < 0.05). Multiple linear regression method was used to construct multiple linear regression equation to explore the effect of FFMI and PhA on FEV1%pred. As shown in Table 8, in multivariate linear regression analysis, PhA was the significant determinants of FEV1%pred.

**Table 7:** Correlation between disease severity and body composition in patients with COPD.

Variable	FEV1%pred		mMRC		CAT		Previous COPD AEs (exacerbations/year)	
	*r*	*P*	*r*	*P*	*r*	*P*	*r*	*P*
Height, m	−0.122	0.215	−0.026	0.796	−0.154	0.116	−0.008	0.937
Weight, kg	0.099	0.314	−0.298	0.002	−0.364	<0.001	−0.024	0.805
BMI, kg/m^2^	0.066	0.502	−0.304	0.002	−0.320	0.001	−0.044	0.653
FFM, kg	0.110	0.265	−0.363	<0.001	−0.447	<0.001	−0.103	0.295
FFMI, kg/m^2^	0.241	0.013	−0.492	<0.001	−0.507	<0.001	−0.143	0.145
PhA, °	0.276	0.004	−0.369	<0.001	−0.455	<0.001	−0.159	0.111
ICW, L	0.110	0.262	−0.370	<0.001	−0.462	<0.001	0.105	0.259
ECW, L	0.082	0.407	−0.353	<0.001	−0.415	<0.001	−0.080	0.414
TBW, L	0.103	0.294	−0.363	<0.001	−0.443	<0.001	−0.102	0.301
ECW/TBW	−0.2977	0.002	0.232	0.017	0.311	0.001	0.175	0.075
ECW/ICW	−0.316	0.001	0.223	0.022	0.304	0.002	0.182	0.063

BMI, body mass index; FFM, fat free mass; FFMI, fat free mass index; PhA, angle of phase; ICW, intracellular water; ECW, the extracellular water ratio; TBW, total body water; FEV1, forced expiratory.

**Table 8:** The factors associated with FEV1%pred based on the multiple linear regression test.

Variable	*b*	*SE*	*t*	*p*
FFMI, kg/m^2^	0.842	0.999	0.842	0.401
PhA, °	5.501	2.757	1.995	0.049

FFMI, fat free mass index; PhA, angle of phase.

## 4 Discussion

Due to systemic inflammatory response, hormonal disorders, hypoxia, and anorexia, COPD patients may suffer from malnutrition, which leads to a series of potential adverse consequences such as decreased quality of life, and increased risk of hospitalization, and death (22). Therefore, nutritional assessment and nutritional intervention strategies for COPD patients are very important in the treatment of COPD. Body composition analysis can be applied to assess the whole body nutritional status in patients with COPD (23). Among them, BIA, which has the advantages of simplicity, accuracy, and reproducibility, is the most widely used method for assessing body composition in patients with COPD in clinical studies (24).

Weight loss is a common body composition change in patients with COPD (25). Mortality is higher in low-body-weight patients with COPD compared with normal-weight individuals (26). BMI is commonly used as a clinically recognized criterion for the diagnosis of malnutrition (12), and a decrease in BMI in patients with COPD has long been established (27). As a marker of malnutrition in patients with COPD (22), BMI has also been used as an objective indicator to assess prognosis and outcomes (12). In this study, patients with COPD showed decreased BMI compared with the control group. In recent years, researchers have found that BMI has uncertainty in identifying malnutrition in patients with COPD (28), because BMI cannot accurately determine whether “obesity” is caused by increased adipose tissue or “wasting” is caused by muscle depletion in patients with COPD (16). Therefore, it does not accurately reflect the changes in body composition.

Studies have further found that patients with COPD frequently have muscle atrophy and decreased FFMI (29). It was shown that even when patients had increased or normal BMI, there was still a decrease in FFMI (16). Consistent with these findings, the present study also showed that FFMI was decreased in patients with COPD compared with the control group. The mechanisms of muscle mass reduction and muscle dysfunction in patients with COPD are not fully understood, and are mainly related to inflammatory activity, oxidative and nitrative stress, hypoxia and hypercapnia, blockade of neuromuscular transmission by smoking, and glucocorticoid use (30). In addition, it has been found that changes in BMI and FFMI are closely related to the severity of airflow limitation in patients with COPD: the BMI of patients with an extremely severe degree of airflow limitation is significantly lower than that of patients with a moderate/severe degree, which may be due to the fact that patients with higher BMI have better nutritional status and higher muscle mass, and can do more exercise to further increase their respiratory muscle content, thus improving their lung function (12). In this study, there was no significant difference in BMI and no correlation between BMI and FEV1%pred between the mild/moderate and severe/very severe airflow limitation groups, whereas FFMI was significantly higher in the mild/moderate group than in the severe/very severe group, and there was a significant positive correlation between FFMI and FEV1%pred, which suggests that FFMI is more useful for evaluating the severity of pulmonary function in COPD compared with BMI (31). In addition to lung function, changes in FFMI are also closely related to clinical symptoms in patients with COPD. Previous studies have shown a significant negative correlation between FFMI and mMRC scores and CAT scores, which was also verified in this study: reduced FFM and FFMI in patients in the more severe group compared with those with the less severe group, and FFM and FFMI were negatively correlated with mMRC scores and CAT scores. An explanation may be that the reduction of FFMI in patients with COPD will further deteriorate the lung function (32), and at the same time, the impaired lung function will also cause varying degrees of damage to the cellular structure of the respiratory muscles, leading to the aggravation of patients’ hypoxia, the disruption of muscle metabolism, the further reduction of FFMI (33), and the aggravation of clinical symptoms, which will lead to the formation of a vicious circle, and therefore, cause a great impact on the exercise capacity of patients with chronic obstructive pulmonary disease (34). Acute exacerbations of COPD are a major cause of accelerated disease progression and lead to early death. A series of studies have shown that acute exacerbation risk is the most important predictor of future exacerbations in patients with COPD, and that the frequency of acute exacerbations is strongly correlated with body composition (35). It has been claimed that weight loss is an independent risk factor for the occurrence of acute exacerbations (36), and that acute exacerbations are more frequent in patients with COPD who have sarcopenia (37). In addition, acute exacerbation of COPD accelerate the impairment of lung function and metabolic disorders, which further leads to muscle atrophy and muscle dysfunction in COPD patients. The results of this study showed that FFMI was significantly higher in the group of patients who were low-risk for acute exacerbations than in the high-risk group, suggesting that FFMI can be used as a predictor for assessing the risk of acute clinical exacerbations. It is hypothesized from these results that early measurement and timely intervention of FFM and FFMI in patients with COPD may improve patients’ clinical symptoms and prognosis.

An increase in ECW/ICW and ECW/TBW can be considered an indicator of loss of muscle mass (38). ECW/ICW can be used as an independent parameter for assessing weight gain after nutritional therapy in patients with COPD (16). In essence, small changes in muscle mass can be reflected by water distribution; therefore, it is not sufficient to measure FFM in patients with COPD, determining the patient’s cellular hydration status is also necessary. In this study, the ICW of patients with COPD was lower than that of the control group, and the ECW was higher than that of the control group, but the difference was not statistically significant, whereas the ECW/TBW and ECW/ICW of COPD patients were significantly higher than those of the control group (*p* < 0.05), suggesting that ECW/TBW and ECW/ICW are better able to reflect the subtle changes of water in the composition of the human body than ECW or ICW alone. This inference was also confirmed in this study: ECW/TBW was significantly lower in the less symptomatic group and in the low-risk of acute exacerbations group than in the more symptomatic group and the high-risk of acute exacerbations group. Additionally, and there was a significant negative correlation between ECW/TBW and FEV1%pred, and a positive correlation between ECW/TBW and mMRC scores and CAT scores. de Blasio et al. (39) have shown an increase in ECW/ICW in patients with COPD with sarcopenia and a significant negative correlation between FFMI and ECW/ICW. Furthermore, patients with high ECW/TBW have low physical functioning and more severe sarcopenia compared with patients with low ECW/TBW (40). It can be inferred that increased ECW/ICW and ECW/TBW are associated with increased clinical symptoms and disease progression. In summary, cellular hydration status (ECW/ICW, ECW/TBW) is useful to evaluate the severity of disease in patients with COPD accurately and comprehensively. Nutritional interventions with reasonable dietary adjustments in patients with COPD may alleviate clinical symptoms and reduce the risk of acute exacerbations. It has been suggested that long-term reductions in carbohydrate intake may lead to a shift of intracellular water to the outside of the cell, thereby affecting body composition outcomes (41). This provides potential new avenues for intervening in the nutritional status of patients with COPD to improve body composition and thereby improve prognosis.

PhA can also reflect the hydration status of cells. Decreased PhA suggests loss of ICW and excessive water transfer to the extracellular compartment and, therefore, an increase in the ECW/ICW ratio; PhA below 5° suggests a significant decrease in ICW and a significant increase in ECW (42). In this study, PhA was significantly higher in the mild/moderate airflow limitation group and the less symptomatic group than in the group with severe/very severe airflow limitation and the more symptomatic group, and it was positively correlated with the FEV1%pred, and negatively correlated with the mMRC scores and CAT scores, suggesting that a decrease in PhA is a predictor of further deterioration of symptoms and lung function in patients with COPD. Martínez-Luna et al. (43) have reported that PhA is a predictor of FEV1%pred, with a 5.74% increase in FEV1%pred for every 1° increase in PhA. In this study, FFMI and PhA were included in the linear regression equation, and the results showed that FEV1%pred was correlated with PhA, but not with FFMI, indicating that PhA was more sensitive than FFMI in predicting lung function in COPD patients. It has also been found that CAT scores and mMRC scores were significantly higher in patients with low PhA compared with patients with normal PhA in COPD, whereas there was no significant difference in CAT and mMRC scores between patients with low FFMI and those with normal FFMI, suggesting that PhA is more sensitive than FFMI in predicting symptoms in patients with COPD (44). A study by Vitalii et al. (45) showed that the frequency of acute exacerbations of COPD was negatively correlated with FFMI. In contrast, PhA in patients with COPD is mainly affected by FFM, suggesting that PhA is a predictor of acute exacerbation in patients with COPD. In this study, PhA was negatively correlated with the number of acute exacerbations in the previous year, but this was not statistically significant. And Karanikas et al. (46) prospectively followed 76 patients hospitalized for acute exacerbations for a period of 1 year and showed that the number of acute exacerbations over that period was negatively, but not significantly, correlated with PhA. Reason for analysis: it may be related to the small sample size of the study, which needs to be expanded in the future to further observe the relationship between PhA and the risk of acute exacerbations of COPD. The above indicates that PhA is closely associated with the degree of airflow limitation and clinical symptoms in patients with COPD, and can be used as an indicator to assist in the comprehensive assessment of the severity of the disease in patients with COPD.

In this study, we applied the BIA method to measure body composition-related indexes in patients with COPD, and comprehensively assessed the body composition characteristics of COPD patients with different disease severity from the perspectives of degree of airflow limitation, clinical symptoms, and acute exacerbation risk. The results suggest that body composition is closely related to the degree of disease severity. This also provides possible avenues for early nutritional intervention and alleviation of clinical symptoms, as well as improvement of lung function and prognosis in patients with COPD.

This study has some limitations. First, the sample size of this single-center study was limited, and the COVID-19 pandemic also limited the recruitment of patients. Second, this study was a cross-sectional study and only assessed the correlation between disease severity and body composition, without providing a causal relationship. In addition, most of the samples collected were from patients with acute exacerbations, and whether there are differences in body composition between patients with acute exacerbations and those with a stable condition needs to be further verified. In the future, it will be necessary to increase the sample size and conduct longitudinal studies to assess the change in body composition in patients with COPD, and the value of nutritional interventions and rehabilitation training to influence clinical symptoms and prognosis. Many of the above aspects are worthy of further study.

## 5 Conclusion

There are significant differences in body composition between COPD patients and the control group and there are significant differences in body composition between COPD patients with different severity of disease, with correlations between body composition and severity of disease.

## Data availability statement

The original contributions presented in the study are included in the article/supplementary material, further inquiries can be directed to the corresponding author.

## Ethics statement

The studies involving humans were approved by the Ethics Committee of the Affiliated Zhongshan Hospital of Dalian University. The studies were conducted in accordance with the local legislation and institutional requirements. The participants provided their written informed consent to participate in this study. Written informed consent was obtained from the individual(s) for the publication of any potentially identifiable images or data included in this article.

## Author contributions

XJ: Data curation, Writing – original draft, Conceptualization, Investigation. YY: Data curation, Writing – review & editing. GC: Writing – review & editing, Data curation. YS: Methodology, Writing – review & editing, Conceptualization. CL: Data curation, Conceptualization, Writing – review & editing. RL: Data curation, Writing – review & editing. YL: Conceptualization, Writing – review & editing, Data curation, Methodology, Resources. LZ: Conceptualization, Data curation, Methodology, Resources, Writing – review & editing.
